# Hepatopulmonary syndrome: pathophysiological mechanisms and clinical implications

**DOI:** 10.1097/ACO.0000000000001538

**Published:** 2025-06-24

**Authors:** Luigi La Via, Elena Ahrens, Antonio Voza, Manfredi Tesauro, Christian Zanza, Yaroslava Longhitano

**Affiliations:** a Department of Anaesthesia and Intensive Care 1, A.O.U. Policlinico ‘G. Rodolico-San Marco’, Catania, Italy; b Department of Anesthesia, Critical Care and Pain Medicine, Beth Israel Deaconess Medical Center, Harvard Medical School; c Center for Anesthesia Research Excellence (CARE), Beth Israel Deaconess Medical Center, Boston, Massachusetts, USA; d Department of Emergency Medicine, Humanitas University-Research Hospital, Rozzano; e Department of Biomedical Sciences, Humanitas University, Pieve Emanuele, Milan; f Department of Systems Medicine, Geriatric Medicine Residency Program, University of Rome ‘Tor Vergata’, Rome, Italy; g Department of Anesthesiology and Perioperative Medicine, University of Pittsburgh, Pittsburgh, Pennsylvania, USA

**Keywords:** dyspnea, echocardiography, hepatopulmonary syndrome, liver transplantation, liver transplantation, platypnea-orthodeoxia

## Abstract

**Purpose of review:**

Hepatopulmonary syndrome (HPS) is a complication of liver disease that affects up to 30% of patients with cirrhosis and portal hypertension and is associated with a more than doubled risk of mortality. This narrative review aims to summarize the pathophysiology and management of HPS, while highlighting indications, timing, and outcomes of liver transplantation as the sole curative therapy.

**Recent findings:**

While supplemental oxygen therapy and pharmacological strategies help alleviate symptoms of HPS, including dyspnea, platypnea, orthodeoxia, and fatigue imposed on signs of liver dysfunction, transplantation remains the sole curative approach. Liver transplantation triples 5-year survival in HPS patients irrespective of baseline disease severity. While data on the correlation between arterial partial pressure of oxygen-based disease severity stages and pretransplant waitlist mortality remain equivocal, the introduction of a model for end-stage liver disease exception points has prompted improved posttransplant survival in these patients. Animal studies provide evidence for pharmacological treatment including norfloxacin or methylene blue, however, large-scale trials targeting humans are lacking.

**Summary:**

While future studies are warranted to investigate the efficacy of medical and interventional treatment options for HPS, liver transplantation remains the only curative therapy. Transplantation demonstrates excellent outcomes independent of disease severity, while optimal timing requires an individualized approach.

**KEY POINTS**

- Hepatopulmonary syndrome (HPS) is a unique pulmonary vascular complication of liver disease characterized by liver dysfunction, impaired gas exchange, and intrapulmonary vascular dilatations.
- While frequently underdiagnosed, HPS occurs in up to 30% of patients with cirrhosis and portal hypertension and is characterized by dyspnea, platypnea, orthodeoxia, and fatigue.
- Liver transplantation remains the sole curative therapeutical approach, with excellent survival rates and often resolution of HPS within 12 months.
- Timing of transplantation requires a tailored approach, taking into account comorbidities, burden of symptoms, and disease severity.
- Future studies are warranted to investigate medical treatment options, such as norfloxacin and methylene blue, in human studies under controlled conditions.

## INTRODUCTION

Hepatopulmonary syndrome (HPS) is a unique pulmonary vascular complication of liver disease characterized by liver dysfunction, gas exchange abnormalities, and intrapulmonary vascular dilatations [1]. Its prevalence reaches up to 30% in patients with cirrhosis and portal hypertension, depending on applied diagnostic criteria and the studied patient population [2^■■^]. Pulmonary manifestations of liver disease are a well-known phenomenon, with initial reports dating back to 1884 when Flückiger first described a patient with cirrhosis, cyanosis, and digital clubbing [3]. However, the entity of HPS itself was not established until 1977, when Kennedy and Knudson [4] introduced the syndrome to describe the association of hepatic dysfunction with hypoxemia. The perception that liver disease is associated with impaired gas exchange due to anatomic shunts bypassing the pulmonary circulation has shifted under the work of Krowka and colleagues in 1984, who proposed that small intrapulmonary vascular dilatations are the primary mechanism responsible for this impairment in liver disease [5]. Studies in the early 2000s clarified the complex pathogenesis of HPS, which gained particular attention when found to be associated with a more than doubled mortality in 218 liver transplant candidates in a prospective cohort study across seven centers in the USA [6]. Finally, the inclusion of HPS-related parameters into some organ allocation systems corroborated its relevance for transplant indications and outcomes [7]. This narrative review will discuss the pathophysiology, diagnosis, and management of HPS, while highlighting indications, timing, and outcomes of liver transplantation as the sole curative therapy. This work aims to provide clinicians with evidence-based insights into both the underlying mechanisms and management of this syndrome by synthesizing knowledge derived from basic science, practice, and clinical research [8^■■^].

## PATHOPHYSIOLOGY

### Pulmonary vessel dilatation

The key pathomechanism in HPS is dilatation of pulmonary microvasculature, especially at precapillary and capillary levels: Capillary diameters in healthy volunteers range from about 8 to 15 μm, whereas in HPS, diameters of up to 100 μm have been observed [9]. This heterogeneously distributed vessel dilatation can be explained by increased synthesis of nitric oxide (NO) in the context of underlying liver disease [10], which was initially suspected due to reports of more than tripled concentrations of exhaled NO in these patients [11]. Three mediators of increased synthesis of NO, which induce vascular relaxation via the guanylate cyclase signaling pathway [12], have been described:

- Endotoxines: Portal hypertension perpetuates intestinal bacteria translocation, which in turn promotes endotoxinemia [13]. Proinflammatory cytokines, including tumor necrosis factor-alpha (TNF-α), subsequently upregulate the expression of NO [14].
- Endothelin-1 (ET-1): Cirrhosis increases hepatic vascular resistance, resulting in impaired clearance of vasoactive substances (e.g. ET-1). Although ET-1 is a vasoconstrictor, it paradoxically mediates stimulation of NO production in the pulmonary vasculature [15].
- Carbon monoxide: In liver cirrhosis, enhanced heme oxygenase-1 activity increases carbon monoxide production, which potentiates NO-associated vasodilation [16].

### Angiogenesis

HPS involves structural remodeling of the pulmonary vascular bed through angiogenesis, leading to the formation of new, often dilated, blood vessels that bypass normal gas exchange units [17]. Several key mediators drive this angiogenic response. For example, serum vascular endothelial growth factor and placental growth factor levels are elevated in patients with HPS and correlate with disease severity [18]. These factors synergistically promote endothelial cell proliferation and migration, essential steps in new vessel formation [19]. Further, pulmonary intravascular CD68(+)macrophages accumulate in the pulmonary vasculature in experimental models of HPS and produce numerous pro-angiogenic factors. Their crucial role in HPS was first demonstrated when depletion prevented and reversed the histological and hemodynamic features of HPS, including increased medial thickness and obstruction of small pulmonary arteries in 42 sham rats [20].

### Gas exchange abnormalities

The vascular changes in HPS lead to three distinct mechanisms of gas exchange impairment that collectively contribute to chronic hypoxemia:

- Ventilation-perfusion (V/Q) mismatch: Dilatation of pulmonary vessels in the presence of normal alveolar ventilation creates areas of low V/Q ratio, resulting in inadequate oxygenation of blood flowing through these units. This is the predominant mechanism in mild to moderate HPS and explains the typical response to supplemental oxygen therapy observed in the majority of these patients [21].
- Intrapulmonary shunting: In severe HPS, true anatomic shunts develop from direct arteriovenous communications, allowing blood to bypass ventilated alveoli entirely. These shunts represent functional right-to-left communications and contribute to hypoxemia that is minimally responsive to oxygen supplementation [22].
- Diffusion limitation: The increased diameter of pulmonary capillaries creates a greater diffusion distance for oxygen molecules, while increased cardiac output common in cirrhosis reduces the transit time of erythrocytes through the pulmonary circulation, further impairing oxygen uptake. This mechanism is quantified by the alveolar-arterial oxygen gradient (A-a gradient) and explains the characteristic orthodeoxia (decrease in PaO_2_ of >5% or >4 mmHg when moving from supine to upright position) observed in HPS, as gravity increases blood flow to dilated vessels in dependent lung regions [23].

The relative contribution of these mechanisms varies among patients and correlates with disease severity. Early HPS typically presents with predominant V/Q mismatch, while advanced cases develop significant shunting and diffusion limitation [24–29].

## DIAGNOSTIC CRITERIA AND CLASSIFICATION

HPS is frequently underdiagnosed in patients with liver disease, with one retrospective study on 42 749 individuals with cirrhosis demonstrating a prevalence of International Classification of Diseases diagnoses of only 0.5% [30]. These findings stress the importance of standardized screening in these patients. The diagnosis of HPS is based on a triad comprising liver disease or portal hypertension, impaired oxygenation, and evidence of intrapulmonary vascular dilatations [31]. For purposes of diagnoses, gas exchange abnormality is defined as an A-a gradient ≥15 mmHg in patients when breathing air at sea level (≥20 mmHg in patients older than 65 years of age) by the European Respiratory Society Task Force on Pulmonary-Hepatic Vascular Disorders [32]. The presence of intrapulmonary vascular dilatations is detected using contrast-enhanced echocardiography as the screening modality of choice due to its high sensitivity, noninvasiveness, and broad availability [33]. Other techniques consist of 99mTc-MAA (technetium-99 m-labeled macroaggregated albumin lung perfusion scanning), which indirectly quantifies intrapulmonary vascular dilatations by measuring the proportion of the radiotracer that is shunted away from the pulmonary circulation and deposited in extrapulmonary sites, including the brain [34]. Notably, two types of HPS have been described in literature based on the predominant location of pulmonary vessel dilatation. Type I HPS is characterized by dilatation of vessels at the precapillary level, while Type II is anatomic shunting via direct arteriovenous communications [35–40]. This differentiation has therapeutic implications, as Type I, but not Type II classically responds to supplemental oxygen [41].

### Severity

Severity classification provides important prognostic information and informs management decisions, especially in relation to prioritizing liver transplantation. The grading of HPS severity relies predominantly on the level of oxygenation impairment, expressed as partial pressure of oxygen (PaO_2_) values when breathing room air at sea level [32]. A four-tier classification of HPS is the most widely accepted, with cutoffs at PaO₂ ≥80 mmHg, ≥60 to <80 mmHg, ≥50 to <60 mmHg, and <50 mmHg corresponding to mild, moderate, severe, and very severe disease, respectively (Table 1) [33]. Data on the correlation between severity stages and pretransplant waitlist mortality remain equivocal, with one study reporting an association of PaO_2_ with transplant timing (*P* = 0.007) but not with death on the waiting list (*P* = 0.33), further highlighting the need for tailored decision-making [42]. Prognostic implications have also influenced changes in organ allocation policy: Generally, patients with HPS and PaO₂ <60 mmHg apply for model for end-stage liver disease (MELD) exception points in the USA [43], acknowledging an elevated risk of mortality unrelated to hepatic synthetic dysfunction.

**Table 1.** Disease stages of hepatopulmonary syndrome

Stage	P[A-a] O_2_ (Alveolar-arterial oxygen gradient)	PO_2_ (partial pressure of oxygen)
Mild	≥15 or ≥20 mmHg in patients aged >64 years old	≥80 mmHg
Moderate		≥60 to < 80 mmHg
Severe		≥50 to < 60 mmHg
Very severe		<50 mmHg

## CLINICAL MANIFESTATIONS

**FIGURE 1.**
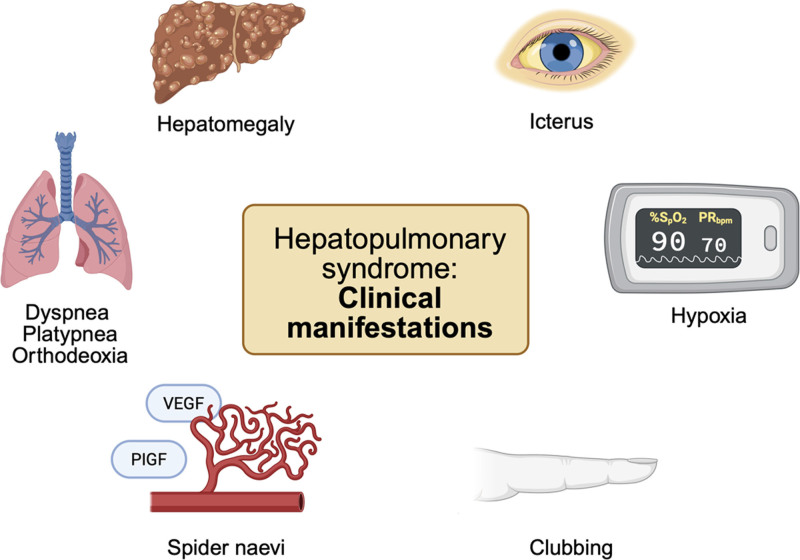
Clinical manifestations of hepatopulmonary syndrome. PIGF, placental growth factor; VEGF, vascular endothelial growth factor.

HPS manifests with respiratory and systemic symptoms superimposed on signs of liver disease. While asymptomatic cases occur, the cardinal symptom, dyspnea, occurs in approximately 80% of cases [44], and often leads to marked functional limitation in advanced disease stages. Most patients further experience platypnea, an aggravation of dyspnea in upright position with relief when in recumbent position, due to the gravitational redistribution of blood flow to open vessels in the bases of the lungs [45]. Phenotypical characteristics indicative of chronic hypoxemia include digital clubbing, cyanosis, and spider naevi [46]. Fatigue can occur in up to 85% of HPS patients [46], often restricting activities of daily living that exceed what would be expected from their liver disease alone. Lastly, spider naevi as a dermatological stigma of liver disease has been linked to a more than doubled incidence of HPS [31]. Common signs and symptoms of HPS are summarized in Fig. 1.

## MEDICAL MANAGEMENT

While liver transplantation remains the only effective, yet radical treatment of HPS, medical therapies and supportive measures play crucial roles in symptom management, quality of life improvement, and bridge-to-transplant optimization.

Supplemental oxygen therapy is the foundation of symptomatic management for patients with hypoxemia associated with HPS [47]. It increases alveolar oxygen tension, improving diffusion across the disproportionately long diffusion path generated by dilated pulmonary vessels, and enhancing arterial oxygenation. The initiation of oxygen therapy should follow defined criteria, although practices vary. Indications are generally accepted as resting PaO₂ <90% [48].

Numerous pharmacological interventions have been investigated for HPS, targeting various elements of its complex pathophysiology. While no medical therapy has consistently achieved reversal of the syndrome, several agents show potential benefits and remain subjects of ongoing research. First, NO inhibitors have attracted interest given NO’s central role in pulmonary vasodilation in HPS. Methylene blue, a guanylate cyclase inhibitor, has demonstrated improvements in PaO_2_ (58 to 74 mmHg; *P* = 0.006), shunt fraction (41 to 25%; *P* < 0.001), and cardiac output (10.6 to 8.6 l/min; *P* = 0.008) following intravenous administration at 3 mg/kg over a 15-min period [49]. However, its short half-life of less than 6 h limits practical application [50]. NO synthase inhibitors such as L-NAME show promise in experimental models but remain investigational due to concerns regarding systemic hypertension [51]. Next, TNF-α-targeting agents have theoretical merit based on this cytokine’s role in promoting pulmonary angiogenesis and vasodilation. Pentoxifylline improved gas exchange in small uncontrolled studies [52], but a randomized controlled trial showed no benefit compared to placebo, with gastrointestinal side effects limiting tolerability [14,53,54]. More selective TNF-α inhibitors have shown efficacy in animal models but lack substantial clinical evidence in humans. Lastly, antimicrobial agents targeting bacterial translocation have been explored based on the hypothesis that endotoxemia contributes to pulmonary vascular changes. Norfloxacin has been demonstrated to decreased Gram-negative bacteria translocation from 70 to 0% and the percentage of pulmonary microvessels containing more than 10 macrophages by 50% in rats with common bile duct ligation-induced cirrhosis [55]. A pilot randomized clinical trial of norfloxacin (400 mg 4 times daily over 1 month) could however not reproduce these findings in humans with large variability in disease severity [56], warranting large-scale, well-designed trials to further investigate the efficacy of norfloxacin under standardized conditions.

For a subset of patients with discrete arteriovenous malformations rather than diffuse microvascular dilatation, namely patients with Type II HPS, transcatheter embolization offers a targeted therapeutic approach. Case reports describe successful embolization with improved oxygenation, particularly valuable as a bridge to transplantation or as a palliative concept in severe, refractory hypoxemia [19]. Transjugular intrahepatic portosystemic shunt implantation has yielded mixed results in case reports, with some showing improvement in symptoms and others demonstrating worsening hypoxemia [6]. The theoretical basis for potential benefits includes reducing portal hypertension, thereby lowering the risk of hemorrhage and ascites, and decreasing translocation of gut-derived mediators that stimulate pulmonary vasodilation.

## LIVER TRANSPLANTATION IN HEPATOPULMONARY SYNDROME

Liver transplantation represents the only definitive therapy for HPS, driven by its ability to address the fundamental pathophysiological derangements that perpetuate pulmonary vascular dilatation [57]. In a case-control Kaplan–Meier survival analysis, liver transplantation improved HPS 5-year HPS survival from 23% in those without the transplantation to 76%, irrespective of baseline PaO_2_, cerebral uptake of 99mTc-MAA, or measures of hepatic dysfunction [58].

### Indications

The presence of HPS independently constitutes an indication for liver transplantation evaluation, regardless of underlying liver disease severity [59], acknowledged by both the European Association for the Study of the Liver and the American Association for the Study of Liver Diseases [60]. The decision to transplant in HPS balances several considerations, including hypoxemia severity, progression rate, functional impairment, and underlying liver disease characteristics. Moderate to severe HPS (PaO₂ <60 mmHg) HPS warrants active pursuit of transplantation given the associated mortality risk and likely progression [61], while mild HPS (PaO₂ 60 to 80 mmHg) merits transplant evaluation when associated with functional limitation or evidence of progression. Several factors modify the indications for liver transplant: Rapid oxygenation deterioration (PaO₂ decline >5 mmHg within 6–12 months) suggests aggressive disease that may quickly progress to life-threatening hypoxemia [62]. Evidence suggests certain etiologies, particularly hepatitis C virus infection and alcoholic liver disease, may associate with more rapid HPS progression [63], while cholestatic liver diseases may exhibit more stable HPS courses.

### Timing

Recognition of the unique mortality risk associated with HPS has led to specialized allocation policies worldwide, acknowledging that standard liver dysfunction metrics may not accurately reflect urgency in this population [64]. In the USA, the MELD allocation system includes provisions for MELD exception points for HPS patients, facilitating earlier transplantation access. Current policy grants standardized exception points equivalent to a MELD score of 22, with additional points added every 3 months for candidates who continue to meet the above-described diagnostic criteria [65]. Similar prioritization mechanisms exist internationally. The optimal transplantation timing remains controversial, particularly for very severe hypoxemia (PaO₂ <50 mmHg) [66]. Historical concerns about excessive perioperative mortality in this subgroup led to reluctance to transplant such patients [67]. Data from a 2013 study on 49 HPS patients have however demonstrated no difference in up to 10-year posttransplant survival between groups based on baseline partial pressure of arterial oxygen obtained at the time of HPS diagnosis, leading to more liberal approach to transplantation in these patients [68]. Current approaches favor transplantation before extreme hypoxemia and end-organ damage develop, while underlying liver disease severity, comorbidities, functional status, and center-specific outcomes all contribute to the comprehensive assessment informing optimal timing.

## OUTCOMES AND RESOLUTION OF HEPATOPULMONARY SYNDROME

The long-term prognosis following successful transplantation for HPS is excellent, with complete resolution in most patients at 6–12 months [69]. Resolution of intrapulmonary vascular abnormalities after successful transplantation follows a relatively consistent temporal pattern and is usually assessed objectively through serial measurements of arterial blood gas. One proposed algorithm recommends assessment by arterial blood gas 1, 3, 6, and 12 months after transplant [67]. Initial oxygenation improvement typically occurs within the first few weeks to months posttransplantation, reflecting functional changes in vascular tone rather than structural remodeling [70]. Complete arterial oxygenation normalization may require up to 12 months in most patients, corresponding to the time needed for substantial vascular remodeling and resolution of dilated capillary beds. Contrast echocardiography confirms this timeline, showing gradual reduction and eventual disappearance of intrapulmonary shunting [49]. Recent series report 1-year survival rates of up to 90% and 5-year survival rates ranging between 70 and 80%, comparable to non-HPS liver transplant recipients [68]. Of note, a 2021 systematic review demonstrated that the 5-year survival probability was higher in the post-MELD era (73 vs. 87%; *P* = 0.008), which started in 2002 [69]. While this improvement in survival may be attributable to due to advances in perioperative management over the past 20 years, the implementation of MELD exclusion criteria may also have played a crucial role in the observed survival benefits.

## CONCLUSION

HPS is a unique pulmonary vascular complication of liver disease characterized by liver dysfunction, impaired gas exchange, and intrapulmonary vascular dilatations. Complex mechanisms are involved in disease development and progression, such as the dysregulation of NO, the process of angiogenesis, and the alteration of pulmonary vascular tone. Although supportive therapies provide symptomatic relief, the only curative approach is liver transplantation; outcomes after transplantation have improved substantially in the most recent decade owing to developments in perioperative management and changes in transplant prioritization policies. Following successful transplantation, most patients demonstrate complete resolution of HPS, although recovery rates vary depending on pretransplant disease severity. Large-scale, randomized controlled trials are warranted to investigate medical treatment options and further standardize timing and indications of liver transplant for HPS patients, ultimately leading to better outcomes.

## Acknowledgements

We extend our deepest gratitude to the esteemed Italian-Americans, Leonardo Domiziano Zanza and Lucrezia Flavia Zanza, for their invaluable assistance in revising and editing our manuscript.

## Financial support and sponsorship

None.

## Conflicts of interest

There are no conflicts of interest

## References

[1] Raevens S Boret M Fallon MB. Hepatopulmonary syndrome. JHEP Rep 2022; 4:100527. 36035361 10.1016/j.jhepr.2022.100527 PMC9403489

[2■■] Zaka AZ Mangoura SA Ahmed MA. New updates on hepatopulmonary syndrome: a comprehensive review. Respir Med 2025; 236:107911. 39662637 10.1016/j.rmed.2024.107911

[3] Flückiger M. Vorkommen von trommelschlägelähnlichen Fingerendphalangen ohne chronische Veränderungen an den Lungen oder am Herzen. Wien Med Wochenschr 1884; 34:1457–1458.

[4] Kennedy TC Knudson RJ. Exercise-aggravated hypoxemia and orthodeoxia in cirrhosis. Chest 1977; 72:305–309. 891282 10.1378/chest.72.3.305

[5] Fallon MB Krowka MJ Brown RS; Pulmonary Vascular Complications of Liver Disease Study Group. Impact of hepatopulmonary syndrome on quality of life and survival in liver transplant candidates. Gastroenterology 2008; 135:1168–1175. 18644373 10.1053/j.gastro.2008.06.038 PMC2824882

[6] Swanson KL Wiesner RH Krowka MJ. Natural history of hepatopulmonary syndrome: impact of liver transplantation. Hepatology 2005; 41:1122–1129. 15828054 10.1002/hep.20658

[7] DuBrock HM. Portopulmonary hypertension: management and liver transplantation evaluation. Chest 2023; 164:206–214. 36649754 10.1016/j.chest.2023.01.009

[8■■] DuBrock HM Savale L Sitbon O. International Liver Transplantation Society Practice Guideline Update on portopulmonary hypertension. Liver Transpl 2025; 3:25. doi: 10.1097/LVT.0000000000000600. 10.1097/LVT.0000000000000600 PMC12799263 40094355

[9] Rodriguez-Roisin R Roca J Agusti AG. Gas exchange and pulmonary vascular reactivity in patients with liver cirrhosis. Am Rev Respir Dis 1987; 135:1085–1092. 3579008 10.1164/arrd.1987.135.5.1085

[10] Cremona G Higenbottam TW Mayoral V. Elevated exhaled nitric oxide in patients with hepatopulmonary syndrome. Eur Respir J 1995; 8:1883–1885. 8620957 10.1183/09031936.95.08111883

[11] Rolla G Brussino L Colagrande P. Exhaled nitric oxide and oxygenation abnormalities in hepatic cirrhosis. Hepatology 1997; 26:842–847. 9328302 10.1053/jhep.1997.v26.pm0009328302

[12] Giamello JD Savioli G Longhitano Y. The role of acetazolamide in critical care and emergency medicine. J Geriatr Cardiol 2024; 21:1085–1095. 39734650 10.26599/1671-5411.2024.11.005 PMC11672352

[13] Zanza C Romenskaya T Thangathurai D. Microbiome in critical care: an unconventional and unknown ally. Curr Med Chem 2022; 29:3179–3188. 34525908 10.2174/0929867328666210915115056

[14] Rabiller A Nunes H Lebrec D. Prevention of gram-negative translocation reduces the severity of hepatopulmonary syndrome. Am J Respir Crit Care Med 2002; 166:514–517. 12186830 10.1164/rccm.200201-027OC

[15] Luo B Liu L Tang L. Increased pulmonary vascular endothelin B receptor expression and responsiveness to endothelin-1 in cirrhotic and portal hypertensive rats: a potential mechanism in experimental hepatopulmonary syndrome. J Hepatol 2003; 38:556–563. 12713865 10.1016/s0168-8278(03)00012-6

[16] Carter EP Hartsfield CL Miyazono M. Regulation of heme oxygenase-1 by nitric oxide during hepatopulmonary syndrome. Am J Physiol Lung Cell Mol Physiol 2002; 283:L346–L353. 12114196 10.1152/ajplung.00385.2001

[17] Zhang J Fallon MB. Hepatopulmonary syndrome: update on pathogenesis and clinical features. Nat Rev Gastroenterol Hepatol 2012; 9:539–549. 22751459 10.1038/nrgastro.2012.123 PMC10963041

[18] Robert F Benchenouf F Ha MN. Placental growth factor modulates endothelial NO production and exacerbates experimental hepatopulmonary syndrome. JHEP Rep 2025; 7:101297. 39980753 10.1016/j.jhepr.2024.101297 PMC11840504

[19] Roberts KE Kawut SM Krowka MJ; Pulmonary Vascular Complications of Liver Disease Study Group. Genetic risk factors for hepatopulmonary syndrome in patients with advanced liver disease. Gastroenterology 2010; 139:130–139. 20346360 10.1053/j.gastro.2010.03.044 PMC2908261

[20] Thenappan T Goel A Marsboom G. A central role for CD68(+) macrophages in hepatopulmonary syndrome. Reversal by macrophage depletion. Am J Respir Crit Care Med 2011; 183:1080–1091. 21148721 10.1164/rccm.201008-1303OC PMC3086745

[21] Lima BL França AV Pazin-Filho A. Frequency, clinical characteristics, and respiratory parameters of hepatopulmonary syndrome. Mayo Clin Proc 2004; 79:42–48. 14708947 10.4065/79.1.42

[22] Krowka MJ Tajik AJ Dickson ER. Intrapulmonary vascular dilatations (IPVD) in liver transplant candidates. Screening by two-dimensional contrast-enhanced echocardiography. Chest 1990; 97:1165–1170. 2331913 10.1378/chest.97.5.1165

[23] Martínez GP Barberà JA Visa J. Hepatopulmonary syndrome in candidates for liver transplantation. J Hepatol 2001; 34:651–657. 11434610 10.1016/s0168-8278(00)00108-2

[24] Krowka MJ Wiseman GA Burnett OL. Hepatopulmonary syndrome: a prospective study of relationships between severity of liver disease, PaO(2) response to 100% oxygen, and brain uptake after (99m)Tc MAA lung scanning. Chest 2000; 118:615–624. 10988181 10.1378/chest.118.3.615

[25] Yang W Hu B Wu W. Alveolar type II epithelial cell dysfunction in rat experimental hepatopulmonary syndrome (HPS). PLoS One 2014; 9:e113451. 25419825 10.1371/journal.pone.0113451 PMC4242631

[26] Roberts KE Fallon MB Krowka MJ; Pulmonary Vascular Complications of Liver Disease Study Group. Genetic risk factors for portopulmonary hypertension in patients with advanced liver disease. Am J Respir Crit Care Med 2009; 179:835–842. 19218192 10.1164/rccm.200809-1472OC PMC2675568

[27] Longhitano Y Zanza C Thangathurai D. Gut alterations in septic patients: a biochemical literature review. Rev Recent Clin Trials 2020; 15:289–297. 32781963 10.2174/1574887115666200811105251

[28] Luo B Liu L Tang L. ET-1 and TNF-alpha in HPS: analysis in prehepatic portal hypertension and biliary and nonbiliary cirrhosis in rats. Am J Physiol Gastrointest Liver Physiol 2004; 286:G294–G303. 14715521 10.1152/ajpgi.00298.2003

[29] Diaz GC O’Connor MF Renz JF. Anesthesia for patients with concomitant hepatic and pulmonary dysfunction. Anesthesiol Clin 2016; 34:797–808. 27816135 10.1016/j.anclin.2016.06.012

[30] Bommena S Gerkin RD Agarwal S. Diagnosis of hepatopulmonary syndrome in a large integrated health system. Clin Gastroenterol Hepatol 2021; 19:2370–2378. 33007510 10.1016/j.cgh.2020.09.050

[31] Rodríguez-Roisin R Krowka MJ Hervé P Fallon MB; ERS Task Force Pulmonary-Hepatic Vascular Disorders (PHD) Scientific Committee. Pulmonary-hepatic vascular disorders (PHD). Eur Respir J 2004; 24:861–880. 15516683 10.1183/09031936.04.00010904

[32] Hoerning A Raub S Neudorf U. Pulse oximetry is insufficient for timely diagnosis of hepatopulmonary syndrome in children with liver cirrhosis. J Pediatr 2014; 164:546–552. 24321540 10.1016/j.jpeds.2013.10.070

[33] Vedrinne JM Duperret S Bizollon T. Comparison of transesophageal and transthoracic contrast echocardiography for detection of an intrapulmonary shunt in liver disease. Chest 1997; 111:1236–1240. 9149575 10.1378/chest.111.5.1236

[34] Abrams GA Jaffe CC Hoffer PB. Diagnostic utility of contrast echocardiography and lung perfusion scan in patients with hepatopulmonary syndrome. Gastroenterology 1995; 109:1283–1288. 7557096 10.1016/0016-5085(95)90589-8

[35] Krowka MJ Dickson ER Cortese DA. Hepatopulmonary syndrome. Clinical observations and lack of therapeutic response to somatostatin analogue. Chest 1993; 104:515–521. 8101797 10.1378/chest.104.2.515

[36] Lee KN Lee HJ Shin WW Webb WR. Hypoxemia and liver cirrhosis (hepatopulmonary syndrome) in eight patients: comparison of the central and peripheral pulmonary vasculature. Radiology 1999; 211:549–553. 10228541 10.1148/radiology.211.2.r99ma46549

[37] Gupta S Tang R Al-Hesayen A. Inhaled nitric oxide improves the hepatopulmonary syndrome: a physiologic analysis. Thorax 2021; 76:1142–1145. 33859047 10.1136/thoraxjnl-2020-216128

[38] Sidali S Borie R Sicre de Fontbrune F. Liver disease in germline mutations of telomere-related genes: prevalence, clinical, radiological, pathological features, outcome, and risk factors. Hepatology 2024; 79:1365–1380. 37934624 10.1097/HEP.0000000000000667

[39] Grilo-Bensusan I Pascasio-Acevedo JM. Hepatopulmonary syndrome: what we know and what we would like to know. World J Gastroenterol 2016; 22:5728–5741. 27433086 10.3748/wjg.v22.i25.5728 PMC4932208

[40] Pascasio JM Grilo I López-Pardo FJ. Prevalence and severity of hepatopulmonary syndrome and its influence on survival in liver transplant candidates. J Hepatol 2014; 60:S307–S308. 10.1111/ajt.12713 24730359

[41] Krowka MJ Cortese DA. Hepatopulmonary syndrome: an evolving perspective in the era of liver transplantation. Hepatology 1990; 11:138–142. 2403962 10.1002/hep.1840110123

[42] Kadry Z Schaefer E Krok K. Excellent outcomes with liver transplantation in hepatopulmonary syndrome across pre-transplant PaO2 spectrum. JHEP Rep 2021; 3:100351. 34604726 10.1016/j.jhepr.2021.100351 PMC8473556

[43] Krowka MJ Dickson ER Wiesner RH. A prospective study of pulmonary function and gas exchange following liver transplantation. Chest 1992; 102:1161–1166. 1395761 10.1378/chest.102.4.1161

[44] Palma DT Fallon MB. The hepatopulmonary syndrome. J Hepatol 2006; 45:617–625. 16899322 10.1016/j.jhep.2006.07.002

[45] Schenk P Schöniger-Hekele M Fuhrmann V. Prognostic significance of the hepatopulmonary syndrome in patients with cirrhosis. Gastroenterology 2003; 125:1042–1052. 14517788 10.1016/s0016-5085(03)01207-1

[46] Younis I Sarwar S Butt Z. Clinical characteristics, predictors, and survival among patients with hepatopulmonary syndrome. Ann Hepatol 2015; 14:354–360. 25864216

[47] Krowka MJ. Hepatopulmonary syndrome: what are we learning from interventional radiology, liver transplantation, and other disorders? Gastroenterology 1995; 109:1009–1013. 7657087 10.1016/0016-5085(95)90416-6

[48] Schenk P Madl C Rezaie-Majd S. Methylene blue improves the hepatopulmonary syndrome. Ann Intern Med 2000; 133:701–706. 11074903 10.7326/0003-4819-133-9-200011070-00012

[49] Kianifar HR Khalesi M Mahmoodi E Afzal Aghaei M. Pentoxifylline in hepatopulmonary syndrome. World J Gastroenterol 2012; 18:4912–4916. 23002364 10.3748/wjg.v18.i35.4912 PMC3447274

[50] Tanikella R Philips GM Faulk DK. Pilot study of pentoxifylline in hepatopulmonary syndrome. Liver Transpl 2008; 14:1199–1203. 18668653 10.1002/lt.21482 PMC2834782

[51] Abrams GA Fallon MB. Treatment of hepatopulmonary syndrome with Allium sativum L. (garlic): a pilot trial. J Clin Gastroenterol 1998; 27:232–235. 9802451 10.1097/00004836-199810000-00010

[52] De BK Dutta D Pal SK. The role of garlic in hepatopulmonary syndrome: a randomized controlled trial. Can J Gastroenterol 2010; 24:183–188. 20352147 10.1155/2010/349076 PMC2852224

[53] Gupta LB Kumar A Jaiswal AK. Pentoxifylline therapy for hepatopulmonary syndrome: a pilot study. Arch Intern Med 2008; 168:1820–1823. 18779471 10.1001/archinte.168.16.1820

[54] Gupta S Faughnan ME Lilly L. Norfloxacin therapy for hepatopulmonary syndrome: a pilot randomized controlled trial. Clin Gastroenterol Hepatol 2010; 8:1095–1098. 20816858 10.1016/j.cgh.2010.08.011

[55] Sonavane AD Bagde A Raut V. Therapeutic coil embolization of dominant shunt in hepatopulmonary syndrome enhances post-liver transplant respiratory recovery. Pediatr Transplant 2020; 24:e13729. 32436643 10.1111/petr.13729

[56] Mancuso A Mancuso F Maffeo M. Successful MARS treatment in severe cholestasis patients: effects on liver function parameters, synthetic values, inflammation and fat-soluble vitamins. Saudi J Gastroenterol 2015; 21:152–157.26021774

[57] Martin P DiMartini A Feng S. Evaluation for liver transplantation in adults: 2013 practice guideline by the American Association for the Study of Liver Diseases and the American Society of Transplantation. Hepatology 2014; 59:1144–1165. 24716201 10.1002/hep.26972

[58] Longhitano Y Iannuzzi F Bonatti G. Cerebral autoregulation in non-brain injured patients: a systematic review. Front Neurol 2021; 12:732176. 34899560 10.3389/fneur.2021.732176 PMC8660115

[59] Kim WR Krowka MJ Plevak DJ. Accuracy of Doppler echocardiography in the assessment of pulmonary hypertension in liver transplant candidates. Liver Transpl 2000; 6:453–458. 10915168 10.1053/jlts.2000.7573

[60] Piccioni A Brigida M Loria V. Role of troponin in COVID-19 pandemic: a review of literature. Eur Rev Med Pharmacol Sci 2020; 24:10293–10300. 33090441 10.26355/eurrev_202010_23254

[61] Taillé C Cadranel J Bellocq A. Liver transplantation for hepatopulmonary syndrome: a ten-year experience in Paris, France. Transplantation 2003; 75:1482–9; discussion 1446. 12792501 10.1097/01.TP.0000061612.78954.6C

[62] Salgia RJ Goodrich NP Simpson H. Outcomes of liver transplantation for hepatopulmonary syndrome: a United States update. Transplant Direct 2018; 4:e354.

[63] Krowka MJ Dickson ER Cortese DA. Hepatopulmonary syndrome. Current concepts in diagnostic and therapeutic considerations. Chest 1994; 105:1528–1537. 8181347 10.1378/chest.105.5.1528

[64] Arguedas MR Abrams GA Krowka MJ Fallon MB. Prospective evaluation of outcomes and predictors of mortality in patients with hepatopulmonary syndrome undergoing liver transplantation. Hepatology 2003; 37:192–197. 12500204 10.1053/jhep.2003.50023

[65] Iyer VN Swanson KL Cartin-Ceba R. Hepatopulmonary syndrome: favorable outcomes in the MELD exception era. Hepatology 2013; 57:2427–2435. 22996424 10.1002/hep.26070

[66] Sulieman BM Hunsicker LG Katz DA Voigt MD. OPTN policy regarding prioritization of patients with hepatopulmonary syndrome: does it provide equitable organ allocation? Am J Transplant 2008; 8:954–964. 18416736 10.1111/j.1600-6143.2007.02124.x

[67] Cosarderelioglu C Cosar AM Gurakar M. Hepatopulmonary syndrome and liver transplantation: a recent review of the literature. J Clin Transl Hepatol 2016; 4:47–53. 27047772 10.14218/JCTH.2015.00044 PMC4807143

[68] Khaderi S Khan R Safdar Z. Long-term follow-up of patients after liver transplantation for hepatopulmonary syndrome: a Mexican experience. Transplant Proc 2014; 46:1503–1506.

[69] Aragon Pinto C Iyer VN Hasan Albitar HA. Outcomes of liver transplantation in patients with hepatopulmonary syndrome in the pre and post-MELD eras: a systematic review. Respir Med Res 2021; 80:100852. 34418867 10.1016/j.resmer.2021.100852

[70] Peter C Hongwan D Küpfer A Lauterburg BH. Pharmacokinetics and organ distribution of intravenous and oral methylene blue. Eur J Clin Pharmacol 2000; 56:247–250. 10952480 10.1007/s002280000124

